# Supplementary figures and images for: A large accessory genome and high recombination rates may influence global distribution and broad host range of the fungal plant pathogen Claviceps purpurea
Source: PLoS One. 2022 Feb 10;17(2):e0263496. doi: 10.1371/journal.pone.0263496 (PMC8830672; doi:10.1371/journal.pone.0263496)

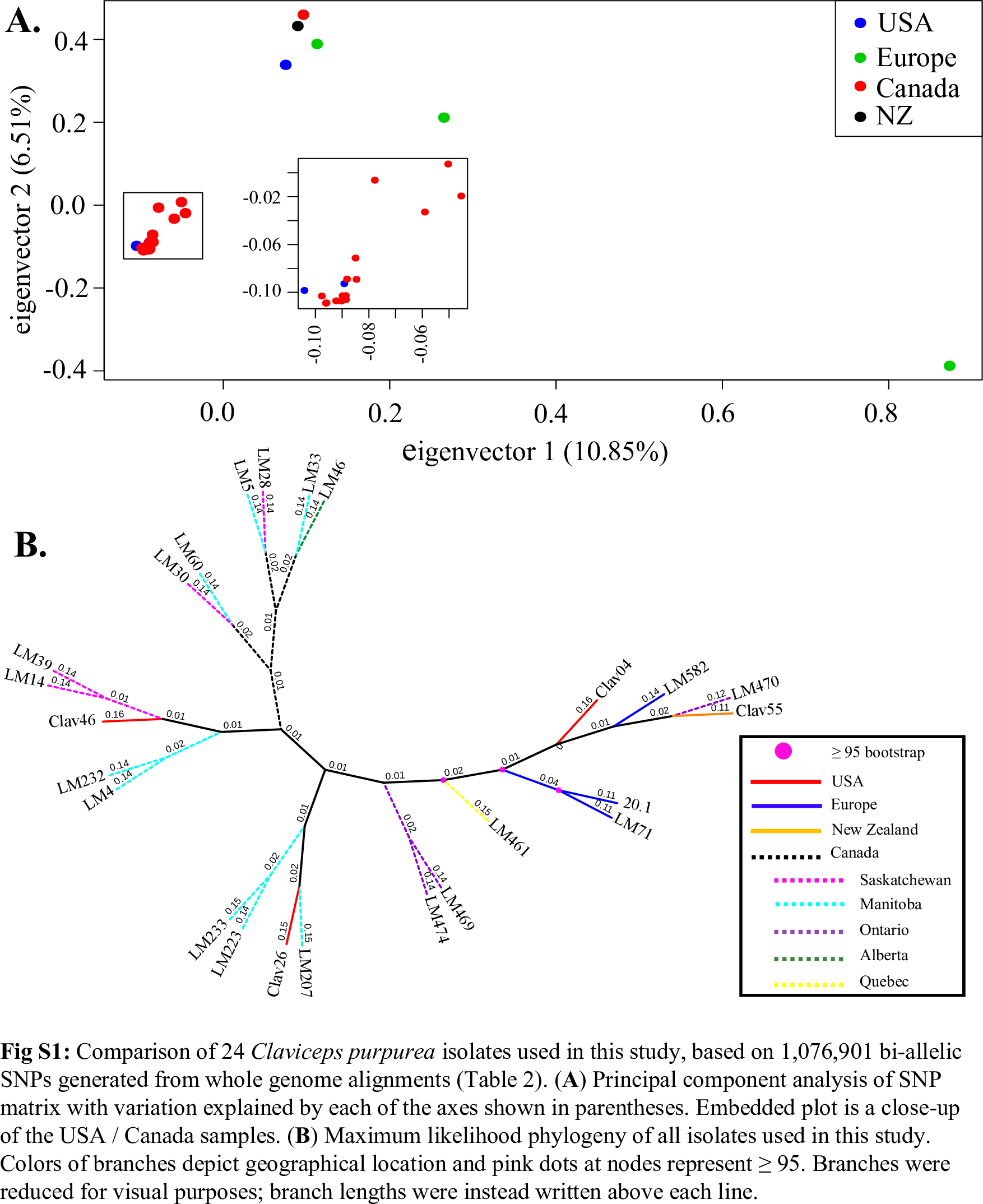

Supplement: S1 Fig — (TIF) [file pone.0263496.s001.tif]

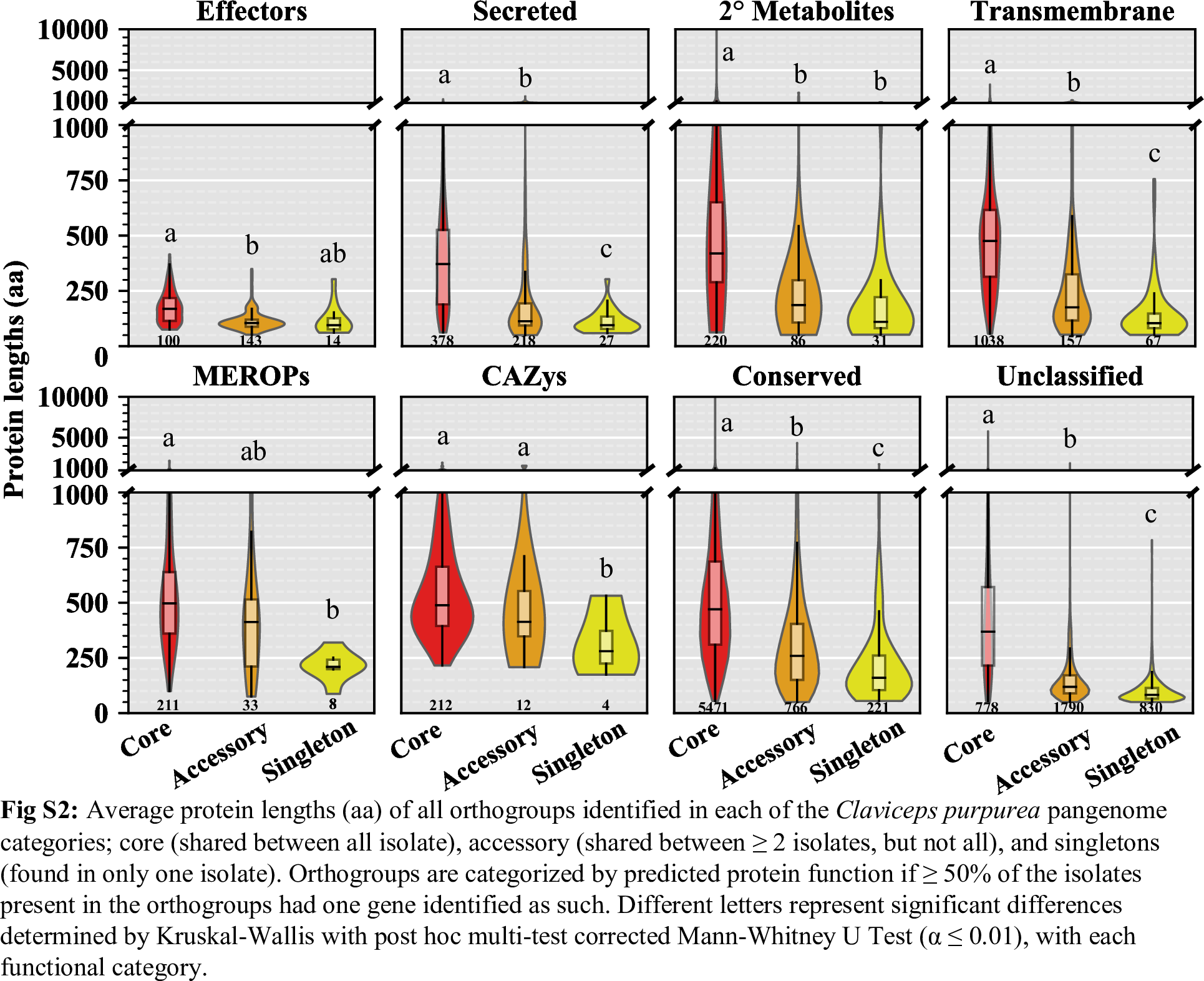

Supplement: S2 Fig — (TIF) [file pone.0263496.s002.tif]

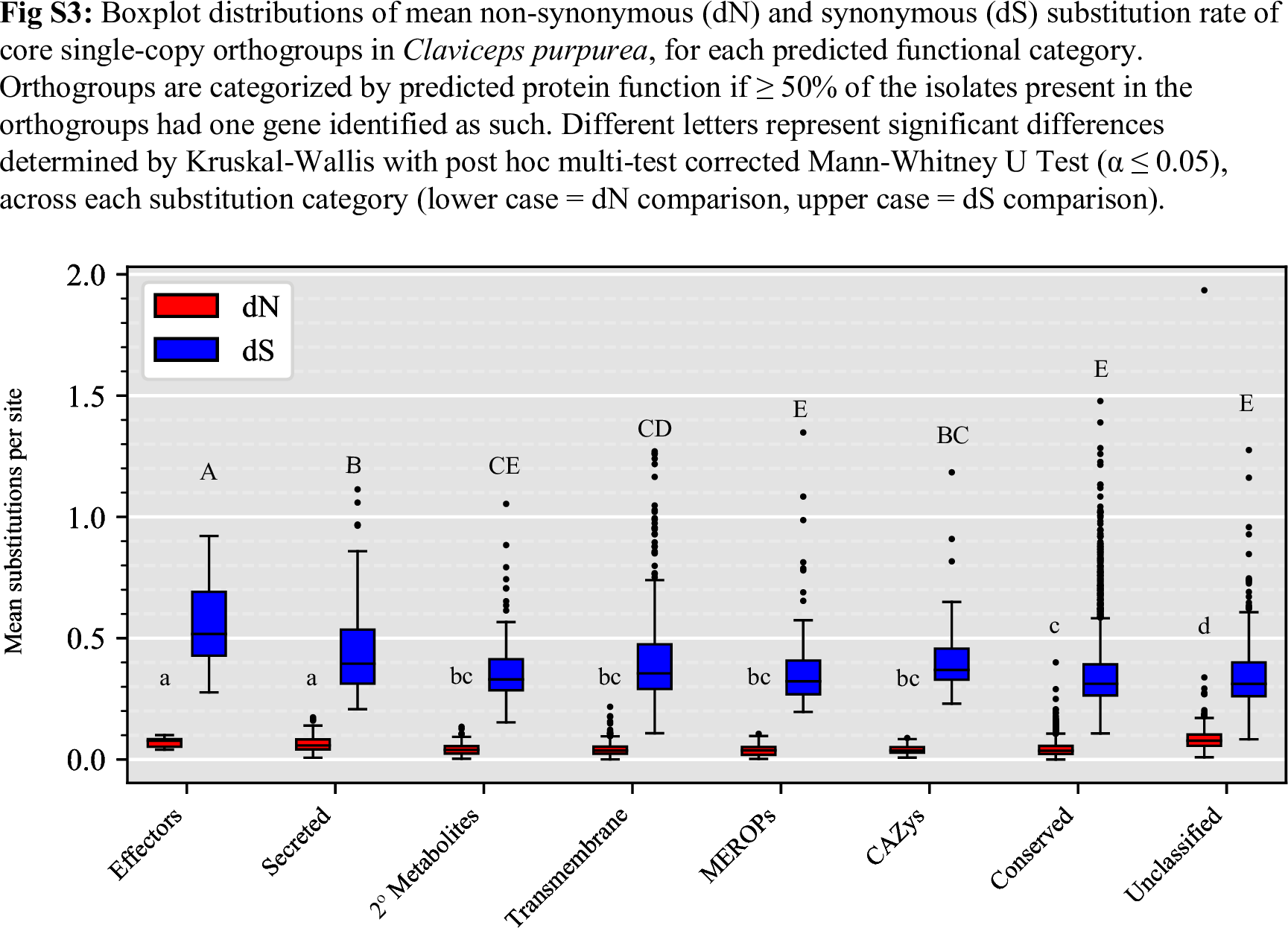

Supplement: S3 Fig — (TIF) [file pone.0263496.s003.tif]

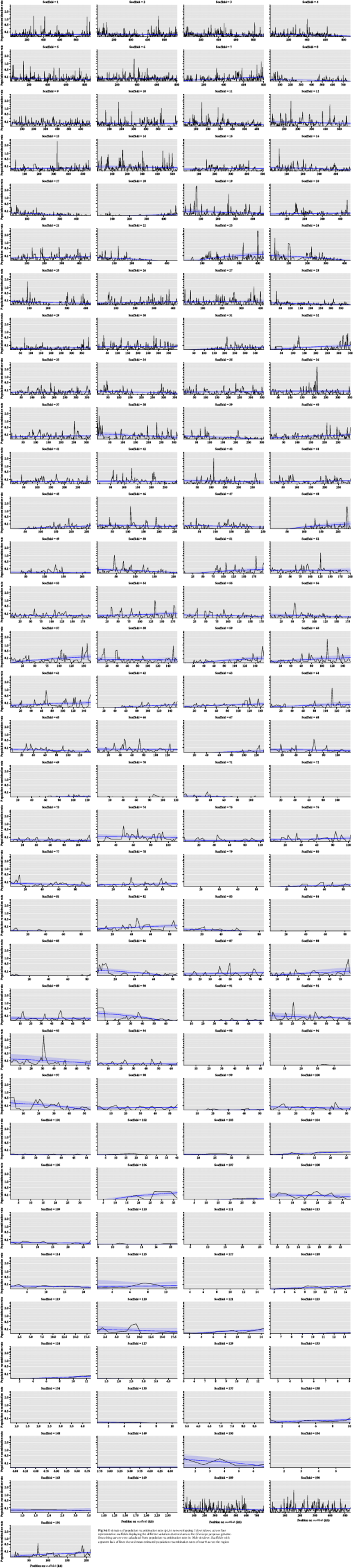

Supplement: S4 Fig — (TIF) [file pone.0263496.s004.tif]

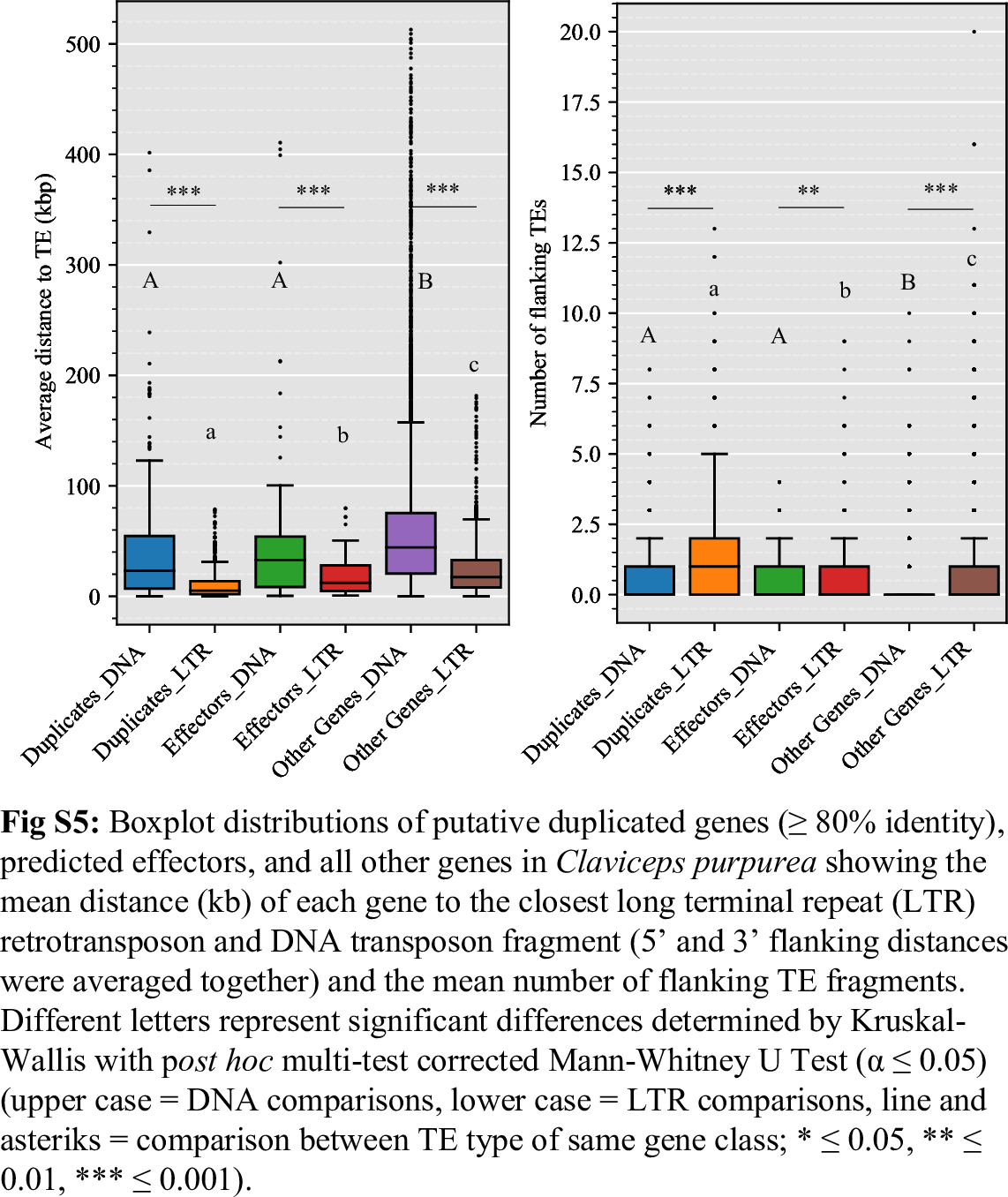

Supplement: S5 Fig — (TIF) [file pone.0263496.s005.tif]

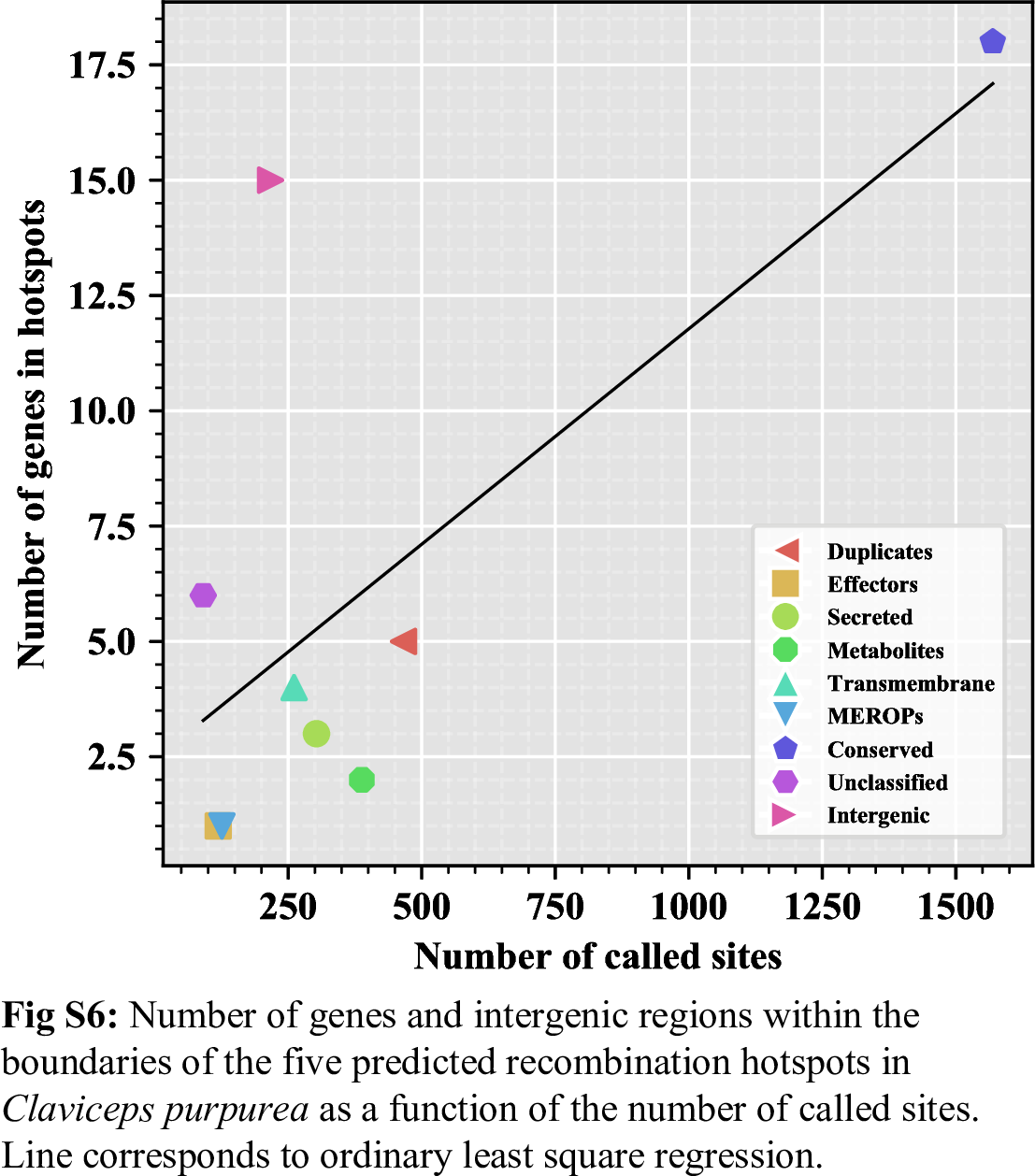

Supplement: S6 Fig — (TIF) [file pone.0263496.s006.tif]

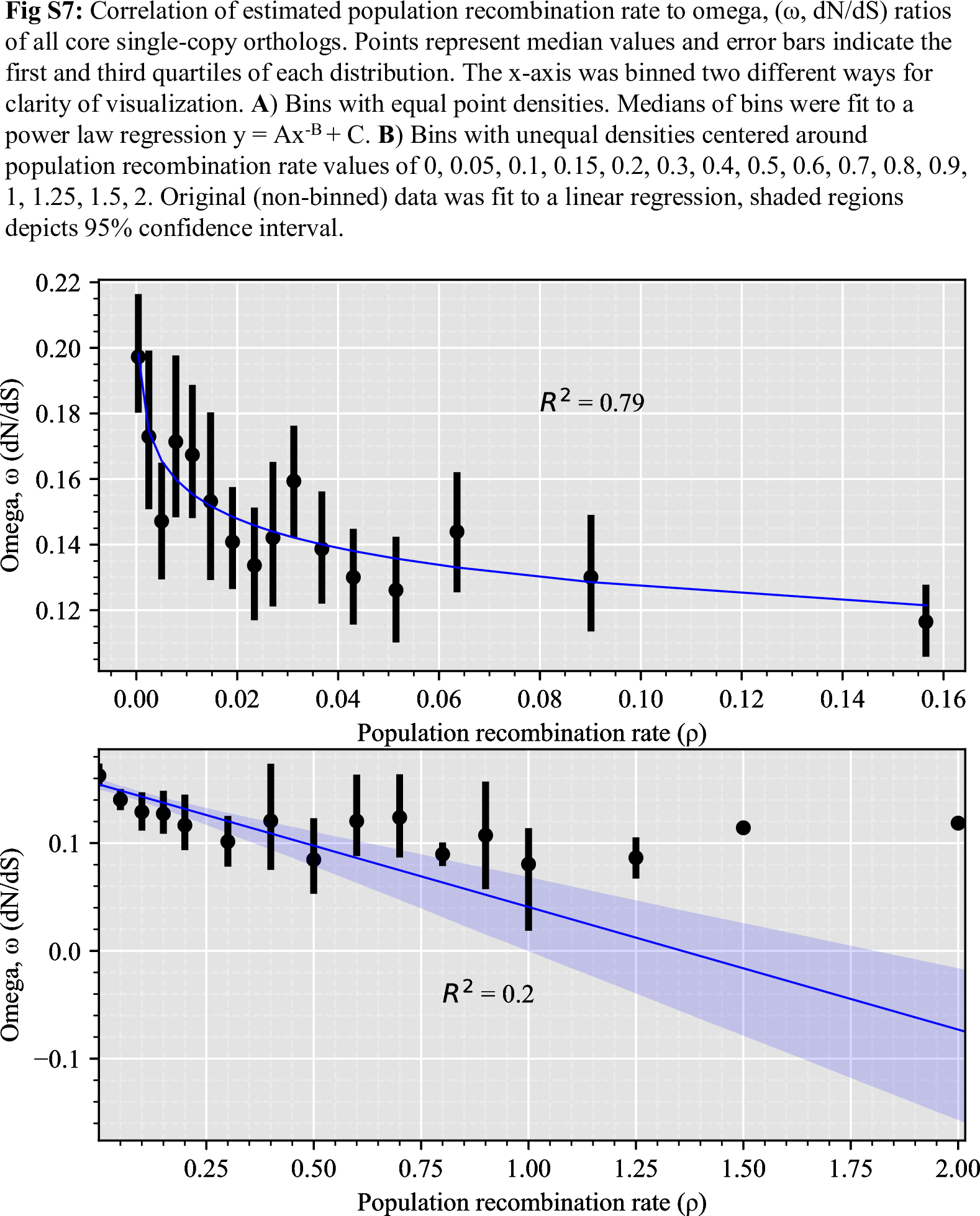

Supplement: S7 Fig — (TIF) [file pone.0263496.s007.tif]
