# Supplementary material for: A large accessory genome and high recombination rates may influence global distribution and broad host range of the fungal plant pathogen Claviceps purpurea
Source: PLoS One. 2022 Feb 10;17(2):e0263496. doi: 10.1371/journal.pone.0263496 (PMC8830672; doi:10.1371/journal.pone.0263496)
Supplement: Attachment — Submitted filename: Reviewers_comments.docx [file pone.0263496.s014.docx]

We thank you for your feedback on our manuscript. We have addressed your comments and have altered the text in accordance with your advice.

Reviewer #1:

**Major concerns:**

252-254: I would be more supportive about the evidence for accessory chromosome(s) if these data were derived from the population-level accessory pangenome. In this study the accessory pangenome references all isolates gathered across the globe – global populations may not exchange genetic material, so 1) How can you distinguish between accessory chromosome scaffolds and genomic regions that evolved from isolation? 2) How can you distinguish them from poorly assembled scaffolds? Short reads are especially prone to not assembling well, potentially leaving streaks of N’s in scaffolds derived from small contigs... particularly from regions with low coverage. At this point, the study points to accessory chromosomes - my assessment is that accessory chromosomes are not substantiated by these data and the other 2 options should be presented as the assumed nulls. – *Claviceps purpurea* is still spread globally through import and export of contaminated grain. In fact, one of the isolates that we have sequenced for this study that was from New Zealand was sent to us by an APHIS agent at a check point in Oregon from an import of contaminated seed. It is very plausible to suggest that not all contaminated seed is identified and that some foreign samples of *C. purpurea* do cross country borders. In addition, Liu et al. 2020 (<https://doi.org/10.1002/ece3.7028>) identified that geographical distribution did not correlate with genetically sub-divided populations of *C. purpurea*. While this section represented a very small portion of our paper, we have further reduced the discussion and presentation of these results and are simply stating a hypothesis that will need to be studied with the use of long-read sequencing and a larger population of samples.

**Other concerns:**

639-646: I’m concerned about the type 1 error thresholds – considering the individual type 1 error thresholds, I think the cumulative type 1 error for the study could be large. The data seems justified visually and corrections are in place in explicit multiple comparisons analyses, but the statistical power of the overall study concerns me. <https://www.ncbi.nlm.nih.gov/pmc/articles/PMC4840791/> - We understand reviewer 1’s concern regarding type 1 error. As noted by the reviewer we have made our best attempt to limit the potential for type 1 error, and the statistical analyses demonstrate this. Given there are multiple analyses in the manuscript it is not possible to measure the statistical power of the entire study. Therefore, we believe we have provided an accurate interpretation of the data, and do not try to over extrapolate these results in the discussion and know there is the potential for type 1 error. Only future experimental data will be able to determine the validity of some of our analyses

Use of normative language without reference points, e.g. vague adjectives as in line 45 “broad genomic analyses”; general grammatic errors/tense switching/active-passive voice switching, e.g. 89 “the factors that were influencing” – We have addressed this throughout the manuscript.

Abstract:
“which is often governed by variable gene content.” - what is governed by variable gene content, what does this mean, what information does this add? - Statement has been removed.

Data locations:
“Dataset, doi: XXX” – remove or add doi – doi has been added

Author Summary:
43-44: “ambiguity surrounding the true nature” is vague, please be specific – Statement has been made more succinct.

Introduction:

*88-89: how does tandem gene duplication implicate their positive contribution to Claviceps – how is this definitively not just a random duplication? Looking for a citation here or explanation of how – Statement has been reworded.

106: “use SCOs to identify genes under positive selection” – the SCOs don’t show which genes are under positive selection, what was the actual method that references the SCOs? – Statement has been altered

Results:
183-184: here, the percentage in ‘()’ is relative to the whole then the next is relative to the previous percentage; previous orthogroup percentages in the text had a common reference point. I think readers could infer, so I can see arguments either way, but I would argue either maintain common reference points or explicitly state them so the reader doesn’t have to infer. – Changed to be consistent with relative to the whole.

185: “two conserved genes” – do you mean orthogroups? – Yes, but these are single-copy orthologs. We believe the designation of gene is appropriate.

224: again, I’m not sure I’d say “genes” when you are referencing what appear to be orthogroups… If there are paralogs within these orthogroups then I don’t think it is appropriate to universally asign a function to the OG because paralogs can take on different functions. At present, it might be appropriate if they are single copy orthologs, but I don’t think that is made clear if that’s the case. – It has been previously stated that the dN/dS analysis and positive selection analysis were only conducted on single-copy orthologs (no paralogs in the orthogroup). Since we are only using single-copy orthologs, we have kept with using the term “gene”

252-254: see major concerns – See comments for major concerns.

*255-270: are these obtained from individual populations or the entire sample set? – Entire sample set.

282: RIP is introduced without expanding the acronym. – Acronym has been expanded

Discussion:
*338-339: how is it supported that this is potentially due to a lack of RIP? – Statement has been removed

417-420: this is not substantiated; perhaps instead of saying “it is plausible to believe”, state the two hypotheses you have mentioned. At present, this is added without any support. - We have altered the text and decided against stating the hypothesis regarding increased mutation due to agricultural practices.

Conclusions:
503-504: This section would benefit from reducing normative language i.e. “large” is used twice, “high” is used - there’s no reference to base these off. Use actual values and if you want to compare with other fungi then actually some sort of reference – Normative language has been toned down.

Methods:
639-646: see general concerns – See comments regarding statistics above.

Reviewer #2: PONE-D-21-02186

A large accessory genome, high recombination rates, and selection of secondary metabolite genes help maintain global distribution and broad host range of the fungal plant pathogen Claviceps purpurea

The authors conducted a comprehensive study on the extended gene repertoire (pangenome) of Claviceps purpurea sampled from different hosts in six countries. The inclusion of samples from different geographical locations and the total number of 24 genomes would be adequate for exploring for the first time the pangenome of this pathogen. Overall, the results presented bring novelty and would be fit for publication in PLOS One after major revisions. Major criticisms are regarding long parts of the discussion being mostly speculations without robust results for supporting their claims. Therefore, the discussion needs to focus on the major novelties presented (i.e., positive selection in secondary metabolites) and shortened in other sections (i.e., mini-chromosome speculation, several comparisons with Zymoseptoria tritici). In my detailed comments below, I request some clarifications and suggest modifications in the manuscript.

The manuscript figures are of high quality, and the authors provide open access to scripts and genomic data, which should be praised.

Specific comments organized according to line numbers in the manuscript:

Results

FigS1: What is the impact of the hosts of origin on the population structure? – This analysis is not a focus of this paper but has been discussed in Miao Liu *et al.* 2020 (<https://doi.org/10.1002/ece3.7028>). In general, *C. purpurea* population genetic subdivisions are not correlated with hosts of origin.

FigS1: Mention the enlarged view within the PCA. - Enlarged PCA has been mentioned.

Line 126: Liu et al not referenced. Would it be possible to provide preprint? – It has since been published and is available https://doi.org/10.1002/ece3.7028. We have updated the reference section.

Line 131: Is 59.2% correct? – Yes, 6,244 / 10,540 = 59.2%. Relative to the whole (pangenome)

Line 151: Please clarify what would be the consequences of unclassified orthologs in the accessory genome for the GO enrichment. – An increased number of unclassified orthologs in the population of the accessory genome will likely increase the type 2 error rate and may mask some potentially enriched GO terms. This caveat has been stated.

Line 200: I understand the details for the “stringent filtering” are in the methods, but briefly mention here. – We have deleted this section, due to removal of the positive selection analysis.

Line 220: Given that effectors in the core genome are not under selection in the present study, how authors see the role of effectors in the accessory genome? – We have deleted this section, due to removal of the positive selection analysis.

Line 252: Since authors used Illumina reads mapped on a not complete (chromosome level) reference genome, how to distinguish whether different scaffolds would ultimately compose a single chromosome and represent indels instead of mini-chromosomes? Also, what would be the impact of structural variations in the pangenome (core + accessory + indels)? – Illumina reads were not mapped to a reference genome. All genomes were assembled *de novo* in Wyka *et al.* 2021, therefore this comment is not pertinent to our study.

Line 283: Why have authors not considered DNA transposons? – We have added DNA transposons to the analysis. The supplemental figure has been updated.

Line 298: “Some hotspots showed a greater association with duplicated genes and TEs (Fig. 7 B-D), while others showed a lower association (Fig. 7 A, E).” ….. If I’m interpreting the figure correctly, I’d suggest being more explicit for Figure 7A = no TE’s within the recombination hotspot. – Text has been made more explicit to follow reviewer’s suggestion.

Discussion

As a general comment, please remove references to tables and figures. – We have received feedback from the editor that is it our choice to remove/keep the references to figures in the discussion. Due to the abundance of figures in the study and supplemental files, we feel that it would improve the ability of the reader to continue to track our discussion with references back to the pertinent figure / data.

Line 363 to 382 is mainly speculative as long reads would be necessary to tackle the mini-chromosome hypothesis. Would it be possible to remove/shorten this section? – This section has been shortened, but we decided to leave it for the purpose of stating the hypothesis which could be important for *C. purpurea* evolution if dispensable are identified by another researcher through the use of long-read sequencing.

Line 324: Zymoseptoria tritici is not a biotrophic fungus (see references: [doi.org/10.1016/j.fgb.2015.04.001and](http://doi.org/10.1016/j.fgb.2015.04.001and) [doi.org/10.1094/phi-i-2011-0407-01](http://doi.org/10.1094/phi-i-2011-0407-01)), and the only grass it infects is wheat (Please see <http://www.genome.org/cgi/doi/10.1101/gr.118851.110> and also Seifbarghi et al 2009). Please, authors should correct this statement and discussion based on this wrong definition. Please clarify that the global distribution of Zymoseptoria tritici is due to wheat infection and that grasses (other than wheat) are infected by different Zymoseptoria.

Line 325: Please tone down comparisons with Zymoseptoria tritici. Z. tritici is heterothallic, has a very limited host range, and develops across a crop season mostly via asexual spores (and hyphae). – (comments for Line 324 and Line 325) We have corrected the statements regarding *Z. tritici* and have altered the discussion around these statements. However, regardless of the fact that *Z. triciti* is only globally distributed because of wheat infections we still feel there is ample claim to compare these two pathogens. *Claviceps* *purpurea* is also a pathogen of wheat as is globally distributed (mostly due to the spread of contaminated seeds between farms). While *C. purpurea* does have a larger host range, both *C. purpurea* and *Z. tritici* are under similar ecological and environmental pressures to survive, which have been shown to influence similar pangenome structures in bacteria.

Line 343: Is there RNAseq data available for improving gene annotation? Considering the signatures of positive selection on unclassified genes, how would positive selection act on pseudogenes in C. purpurea? – The RNAseq data that was available at the time of assembly and annotations (Wyka et al. 2021) was already used in the annotation pipeline to help improve gene predictions. We did specifically look at pseudogenes, but the few pseudogenes that have been found in *C. purpurea* are associated with secondary metabolites, and specifically ergot alkaloids. Gene gains, gene loss and gene sequence changes at the end of chromosomes are believed to be major drivers of ergot alkaloid diversification in the Claviceptaceae (Young et al. 2015). It is possible that many of the many of the unclassified genes are actually psuedogenes that represent remnants of gene loss that have given rise to diverse chemotypes (Young et al. 2015).

Line 428: Populations in areas without fungicide pressure or deployment of resistant cultivars (Israel, Oregon) have similarly high levels of genetic diversity when compared to regions with intensive usage and deployment (Switzerland, Oregon R) as for example in the study of Hartmann et al. 2017. I suggest authors to shorten this discussion section to avoid speculations. – We have removed this discussion point, and left the hypothesis of RIP “leakage”, which is likely a more plausible hypothesis.

Line 435: Are C. purpurea of different hosts able to cross sexually? How would it influence population recombination rates and hotspots analyses? – *C. purpurea* of different hosts are able to cross sexually and do so quite often. This likely increases the effective population size of *C. purpurea* which may inflate recombination rates. However, a larger-scale recombination analysis comparing multiple fungal organisms with LDhot will likely be needed to determine how different lifestyles can influence hotspot analysis. At the time of writing, I only saw *Z. tritici* being analyzed with LDhot.

Line 446: Are high levels of recombination expected in a homothallic fungus? – Sexual recombination has been found to frequently occur in homothallic plant pathogens, such as *Sclerotinia slcerotiorum* (Attanayake et al. 2014, https://www.nature.com/articles/hdy201437) and *Fusarium graminearum* (Talas & McDonald 2015, https://bmcgenomics.biomedcentral.com/articles/10.1186/s12864-015-2166-0), which have large effective population sizes. Certainly, more research needs to be done across additional homothallic fungal species but recombination among homothallic species does appear to occur, but the rate of recombination may depend on the population size and species.

Line 478: I see this section on the evolution of secondary metabolites in C. purpurea as a novel and relevant discussion that should receive focus by shorting previous sections. – This section has largely been removed, due to the removal of the positive selection analysis.

L 488: Reference about Puccinia missing. - The statement was removed from the text due to removal of the CodeML analysis, so no reference is needed.

Materials and Methods

Table 1: I could not find LM28 and 582 in the PRJNA528707. – These genomes are present in PRJNA528707 and have recently been uploaded by NCBI, please see the screenshot of NCBI below.

Line 522: Mention the sequencing technology for the 23 isolates. - Sequencing technology has been mentioned.

Line 524: “cutoff of 50 aa” What does it mean? - Removed “aa” as it’s redundant.

Line 546: What RepBase version? - v24.03

Line 567: Describe how gene sequences of individual genomes were extracted for selection analyses. – Single-copy orthologs from the pangenome analyses (described in the previous section) were used for dN/dS analysis. This has been made more explicit.

Reviewer #3:

The authors use resequenced, assembled, and annotated genomes from 24 C. purpurea isolates to perform a pangenome analysis, and combine this work with two additional analyses, estimating recombination rates (and detecting hotspots), and performing an analysis of selection on coding sequences (single copy orthologous genes). There are many different approaches to infer the action of natural selection in population genetics datasets, and they authors here estimate selection (purifying and positive selection) through estimating dN/dS (omega) for their population set.

The work presented here involves a fairly large dataset, as well as lots of bioinformatics analysis, and so those contributions alone can serve as important resources for understudied species. That said, these large-scale genomic analyses are not that well integrated in this manuscript (at least they are integrated to some extent, especially through various enrichment analyses), and other population genetic analyses could have been included that might help to integrate the different pieces in other ways. The interpretation of population genomic studies is not straightforward, and results tend to be consistent with multiple explanations until more specific analyses are carried out. In this work, the results tend to be over-interpreted, and in many cases the evidence for the authors' conclusions are rather weak. However, unless some important methods descriptions have been left out of this manuscript, the biggest problem in the manuscript is the analysis of natural selection. It appears the authors have carried out the dN/dS analyses with PAML and the CodeML algorithm using only the 24 C. purpurea isolates and without inclusion of data from additional species. The Jeffares et al. paper they cite indicates sequences from multiple species are needed for these analyses [states that PAML FAQ recommends a minimum 4-5 species, refers to multiple alignments of protein-coding gene sequences from several species in a phylogeny, in describing required input files indicates an annotated genome for at least 1 related species is needed besides the target species of interest]. dN/dS analyses are meant to compare nonsynonymous versus synonymous substitutions (alleles gone to fixation in some species or independent lineages), yet what we have in a populations capable of sexual reproduction are segregating polymorphisms (since the isolates are not independent lineages), and the distinction is important. Kryazhimskiy and Plotkin (<https://www.ncbi.nlm.nih.gov/pmc/articles/PMC2596312/>) have shown that inferring selection pressures from dN/dS calculated from population samples does not work in the same way that dN/dS analyses work when multiple species are compared (the behavior of dN/dS is expected to be different). In particular, they find that even for genes experiencing negative selection, the observation of an elevated dN/dS (values closer to 1) is expected with intra-specific samples. And the observation of dN/dS<1 is also consistent with strong positive selection. Given this problem, the major report from the manuscript that secondary metabolism genes are more important than effectors based on the dN/dS analyses must be called into question, since it no longer has any reliable support. – We have incorporated all *Claviceps* species from the Wyka *et al.* 2021 paper and attempted to re-run the analysis on 53 genomes. With the resources on hand we were able to conduct a re-analysis of dN/dS (omega) ratios with YN00, however, the use of the site models in CodeML were too computationally intensive (~24+ hours per single-copy ortholog, ~4,000 in the new dataset) for our current computing capacity. This analysis would require a large expense and time that we currently do not have. Both the lead author (Wyka) and corresponding author (Broders) are no longer at Colorado State University (where the server for the originally analyses was located) and have moved on to different positions respectively, and no longer have access to the CSU server. We do not think the lack of this analysis diminishes the results of this paper or lessens the value of the findings.

The selection analyses represent a major section of the manuscript - lines 174-238 in the Results, 566-588 in the Methods, Figures 3 and 4, Table 2, Supplemental figures S3, S5, S9, and supplemental tables S4-S7 all are based on the highly questionable dN/dS results. To become publishable, the manuscript should either remove the dN/dS analyses (diminishing its findings), or go back and perform the dN/dS analysis using the genome data from multiple species presented in the 2021 GBE Wyka et al paper. – As mentioned above, we have re-done the dN/dS using the *Claviceps* genomes, but have removed the CodeML positive selection analysis.

**Specific comments:**

line 86 - Wyka et al. (2020a) citation - besides updating this reference, make sure it also is listed as 2020a in the Reference list (not just 2000). – Changes have been made.

line 126 - Liu et al. Accepted - This did not appear in the list of references cited. – Correct reference has been added

line 248 – conserved - Done

lines 280-1 - is it possible that there is either bias or some difficulty in estimating the inferred recombination rate associated with duplicated genes (such as potential problems with read mapping and potential genotype errors for these genes)? – For our recombination analysis we used a method that utilizes haploid whole genome alignments with LastZ and MultiZ (Stukenbrock and Dutheil 2018a), so there should be no concerns of errors with read mapping or genotype errors. Unless I am mistaken.

Line 299 - you state genes with conserved domains are most frequent within hotspots - but you are not indicating any overrepresentation, are you? Don't we expect these genes to be most frequent in hotspots because they are most frequent across the whole genome? – Yes, this may be expected, however, we did not compute any enrichment analysis as we only observed 5 recombination hotspots and felt that the statistical power of this test would be very low.

Line 359-60 - Reword, there is not literally a lack of recombination here. - The statement has been reworded.

Lines 447-449 - Your wording seems to indicate that recombination rate is determining whether purifying selection or positive selection is acting, but that is not true. Purifying and positive selection are not mutually exclusive across the genome (one can be happening at some loci, the other at other loci). And high recombination is expected to increase the efficiency of both types of selection. I don't see how high recombination could ever explain few signatures of positive selection. - We agree with your comment, we have altered the text to provide clarity to the statement.

Lines 508-9 - rephrase "likely controlled by" -> likely kept under control, or similar. – Changed to “kept under controlled”.
