# Supplementary material for: A large accessory genome and high recombination rates may influence global distribution and broad host range of the fungal plant pathogen Claviceps purpurea
Source: PLoS One. 2022 Feb 10;17(2):e0263496. doi: 10.1371/journal.pone.0263496 (PMC8830672; doi:10.1371/journal.pone.0263496)
Supplement: Attachment — Submitted filename: 2021-09-22_Reviewers_comments.docx [file pone.0263496.s015.docx]

We thank you for your feedback on our manuscript. We have reviewed your comments and suggested edits and have done our best to address these comments. We have made a number of edits that we believe take into consideration your advice and address the concerns of reviewers.

Reviewer #2: PONE-D-21-02186R1
A large accessory genome and high recombination rates help maintain global distribution and broad host range of the fungal plant pathogen Claviceps purpurea

The authors have addressed all my comments with clear and satisfactory answers. Also, they significantly improved the manuscript by extensively removing and re-writing several parts. After careful reading of this new version, I can recommend it for publication in PLOS ONE.

Thank you for you feedback.

Reviewer #3: The authors have recomputed the dN/dS ratios for their set of gene orthologs, incorporating sequences from species besides C. purpurea, and have removed the analysis of positive selection based on CodeML site models. Those changes have satisfied the main problem I had identified in the original submission. Those edits have a substantial impact on the overall message of the manuscript, and having gone through the revisions, I have identified some other issues that require changes, detailed below.

Tone of manuscript and amount of evidence for conclusions. The strengths of the manuscript come from its scale and breadth (i.e., pan genome, genome-wide, multiple population genomics analyses). However, that breadth comes at a cost of identifying some interesting correlations between population genome features but not being able to demonstrate any strong evidence for causation in these cases. This explains my choice of Partly to answer the question Do the data support the conclusions? There are several general conclusions or statements in the manuscript that suggest causation but are really not well supported by the data and analyses, and these should be revised to tone them down.

Title - Factors X and Y “help maintain global distribution and broad host range” – The title has been altered

Abstract – L26 “likely maintained by high recombination… ”, L28 “likely controlled by frequent recombination” - These statements have been toned down

Intro L98 “likely maintained by” – This statement has been toned down

Certain references to genes under positive selection – see comment below.

Appreciation for low power of whole-gene dN/dS ratio analysis. I have 2 points related to the interpretation of the whole gene dN/dS ratios. 1st) Genes with dN/dS >1 are not necessarily under positive selection, particularly for genes with ratios only slightly greater than 1. Some test would be required to show a particular ratio is significantly greater than 1. It would be better to refer to genes with the ratio >1 as positive selection candidates, or genes with signatures (or evidence) of positive selection (phrases that are used in several places in the manuscript).

The following phrases should be revised:

L96 “identified genes under positive selection,” – This phrase has been re-worded.

L115 “landscape of genes under positive selection”. – This phrase has been re-worded.

2nd) A dN/dS ratio for a whole gene being > 1 is a very stringent criterion for positive selection (that is, at least when it is significantly greater than 1). The literature is full of examples where positive selection is indicated but where the whole gene dN/dS ratio is < 1. That means this analysis has very low power for detected genes with sites under positive selection, and so the absence for evidence of positive selection for a category of genes should not be confused with evidence for the absence of positive selection. When considering factors that can explain no evidence for positive selection on a subset of genes (such as L489-490), the most important one should be this lack of power to detect it. The reviewers make an excellent point and he have incorporated this into the text. – New lines 504-506

Keep in-text reference citations consistent (all numerical, without publication year). As appropriate, most reference citations have been converted to numerical format, but some hold-overs of the (author, year) style remain. For example, L118, “in Wyka et al. (2021)” should be edited to “in Wyka et al. [17]” or even just “in [17].” – We have altered the references to be more consistent. If a reference was at the start of a sentence we decided to go with Wyka et al. [17], for all other cases we used the numerical format.

Minor points:

L112 should also reference [17] – Reference has been added.

L114 Amino acid cutoff requires some context here to be clear (such as the phrase For the purpose of defining gene models for subsequent analysis…). The appropriate context can only be found in the referenced papers currently. – We have altered the sentence to reflect the reviewers feedback.

L162 After filtering for positive selection – this analysis has been removed from the manuscript, correct? Then this sentence needs to go as well. – Sentence has been removed

Gap between L203-204. Something is missing between hotspots and 1000 simulations. – The sentence has been corrected.

L379 selection of secondary metabolite genes – this result is gone from the manuscript, so this needs to be removed here as well. – The statement has been removed

L400 Citation of Fig S7 should be changed to Fig S5. – Has been changed
Table S6 lists 36 scaffolds, but the text refers to 37 missing scaffolds. – Has been corrected to 36 in the text.

Reviewer #4: This study explores the pangenome of Claviceps purpurea, as well as the recombination and selection landscapes based on 24 isolates. It is overall a nice study with interesting new results. I understand that this is a revised version of a previous manuscript, and, although I was not part of the previous reviewing process, it seems that the major methodology flaws pointed out were corrected by the authors. A sizeable part of the original analyses has been left out of the current manuscript entirely as the authors state that they do not have access to the computational power they had when the study was first written. As such I have been careful to make only suggestions for which the data should already be generated.

The introduction is clear and sets up the following text well. The methods section is detailed and scripts are provided publicly. The results are clearly explained. I have some concerns with the discussion which is not yet at a level of clarity as excellent as the rest of the manuscript. I also have some questions regarding the pangenome-related analyses.

Main comments:
One concern with the manuscript as it stands now is that the different sections feel very disconnected from each other, both in the results and in the discussion. The study starts with a description of the pangenome. Then the authors go on looking at selection only on core genes and only look at the recombination, once again, in fragments shared by all samples. I did not find an explanation in the manuscript as to why accessory genes found at a sufficiently high frequency could not be analysed for selection for instance and the results between accessory and core genes then compared. – We provided an additional rebuttal to this comment about accessory genes in the following concerns. To answer the question, “why we didn’t include accessory genes in the selection analysis”, we chose not to do so as previously we only included *C. purpurea* isolates for the dN/dS ratio. This approach was incorrect, so we needed to include other species to provide power to the selection analysis. We did do this by including the remaining species that we had genomes for in the *Claviceps* genus. Choosing to run the selection on only core genes make sense as the dataset will be consistent, and all genes will have been compared to all other *Claviceps* species. If we started to look at accessory genes then we would also reduce the number of *Claviceps* species used in each dN/dS calculation. For example, core genes used alignments from 29 other *Claviceps* species to calculate dN/dS, however, if we start to look at accessory genes then the number of other *Claviceps* species will be reduced and thus would not provide a consistent dataset to compare against the core genes.

Many of the comparisons made to other species are made with Z. triciti which has an accessory genome organized in core chromosomes separated from accessory chromosomes. Some of the comparisons would be more relevant if there were hints of a similar organization in C. purpurea. Here, despite the authors basing their study on de novo assemblies of the genomes, the only attempt to understand the distribution in the genome of core and accessory genes is a comment on fragments that are “missing” from the MGA. It is not clear to me what “missing” implies. Does this mean that the fragments are found in only one isolate and that these are not reported by the alignment tool? Are these the contigs containing the singleton genes? If so, the numbers do not match. Where are the other singleton genes? If they are aligning, would this not point to a possible issue with the way the pangenome was defined in the first place? Please clarify this as this could potentially be a problem. – It is difficult to follow the reviewers statement here as there are no direct references and they do not indicate which “numbers do not match up”. From the Multiple Genome Alignment (MGA) we only kept scaffolds (based on the reference genome) that had alignments from all of the remaining 23 genomes. The reference genome contained 191 scaffold and from the final MGA we kept 154 scaffolds. The scaffolds that were not kept contain mostly accessory and singleton genes (as stated in S6_table). However, there are some cases of core genes being present on these scaffolds. The next question will be “Then how are there core genes on scaffolds that are not part of the scaffolds that were kept”. The answer to this question is that the pangenome was conducted with OrthoFinder (as other pangenome papers have also used), which groups the proteins into orthologous groups (OGs). OGs are then classified as “Core” if they contain proteins from all 24 genomes, regardless of whether the OG is paralogous (i.e. some genomes may contain 2 copies of the gene while others contain 1). If the core OG is paralogous for the reference genome or all but 1 genome, then it is entirely possible that the 1+ genomes that contain only 1 copy of the protein will not have a DNA region to align to the scaffold that contains the 2^nd^ copy. This scaffold would then lack all 24 genomes and not be kept from the MGA. Since only a handful of scaffolds were not kept, and contain a large majority of accessory genes and TEs we did not see any fault in the logic of how the pangenome was created.

As for other accessory and singleton genes, the MGA also contain alignments that were either shared by 1-23 of the other genomes and not the reference. Therefore, these alignments were not kept as they did not map to the reference.

The authors hypothesize that these missing contigs are a hint of possible accessory chromosomes. I (like previous reviewers) am not at all convinced by this argument. Indeed, the abundance of reverse transcriptase and other repeat associated genes on these fragments would point more towards assembly artefacts. – We are stating this more as a potential hypothesis, but have made it more clear in the text that assembly artifacts could also be a likely hypothesis.

However, I do believe that understanding the genomic distribution of the core and accessory regions is extremely relevant to the topic of this study and should be attempted. Where are the accessory genes in the de novo assemblies? Are accessory/singleton genes systematically found together on the same contigs? Are they found more often on the same contigs or do they share contigs with core genes? I believe that the data should already be generated by the authors to answer most of these questions without need for new in-depth analyses. – If we had chromosome level genomes I could understand the reviewers comment here about understanding where accessory genes are located, in comparison to core genes. However, without such data we do not believe the analysis would strengthen the paper, but in fact would provide a discussion on results that do not draw any strong conclusions. Since many of the *de novo* assemblies contained > 1,000 contigs we feel that such an analysis may also lead to inaccurate results as a contig that contains many accessory genes may in fact be flanked by two contigs that contains large amounts of core genes. Without long-read sequencing data we would not be able to accurately examine the true distribution of these genes.

Accessory genomes are frequently hypothesized (and indeed in some cases proven) to play a role in host specificity. I’m surprised that no mention of this was made in the results (perhaps I missed it?). Several of the strains come from the same host species (Hordeum vulgare, for example). Were there accessory genes shared by these strains? Surely, the data is already available easily from the orthogroups table. This also seems like a relevant trait to look for when describing the pangenome of a species with a broad host range, especially as the authors report an enrichment of effectors in the accessory genes. – Single isolates of *Claviceps purpurea* can infect multiple hosts and this has been shown in several different manuscripts over the years. While many isolates may have been isolated from a single host species, we do not believe that shared genes between that snap-shot of isolation will reveal any correlation to an ability to infect *H. vulgare* (for example). In addition, additional work that was sparked from this paper looked at population structure of *C. purpurea* and found that host groups did not show any correlation with population structures (DOI: 10.1002/ece3.7028). To really test this proposed hypothesis we would need to conduct a broad grass inoculation panel of all the *C. purpurea* strains used in this study to truly identify any lack of infection potential and then conduct a correlation analysis of gene content. Currently, we do not have the means and capability to conduct such inoculation trials as I have graduated and do not have funding / resources.

None of these concerns are a cause for stopping the publication of the manuscript even without any new analyses, if appropriate explanations are provided as to why these analyses would be impossible/not relevant to the study.


Minor comments:

As previously noted by the reviewers the discussion contains large section of comparison to Z.tritici and these are not always so relevant that they deserve the word count they get.

L.387: The authors argue that the host distribution of these two species are similar. Z.tritici is strictly a wheat pathogen when the authors state that C. purpurea “has a broad host range of ~ 400 grass species across 8 grass tribes”. Arguing that the hosts are similar enough to lead to a convergent evolution of pangenome size seems to be a stretch. – The statement has been altered to only reference the similar geographical distribution as the factor that may help explain a similarly large accessory genome.

L.407. “Badet et al. suggested that TEs were likely contributing to Z. tritici accessory genome“. In this study, the accessory chromosomes are including non-genic content (based on PacBio genome assemblies), making the TEs having a clear role in expansion of the accessory genome. I am not sure how relevant this comparison is to a pangenome in which only the genes were considered. Perhaps focus this paragraph more on the results obtained in the current study instead of describing in details results from another paper. – From the previous revision we have removed some of the discussion around *Z. tritici*, however, we have decided to leave this paragraph as it does provide a medium and helps convey the results we obtained from *C. purpurea*, as it provided a reference point for comparison with another organism.

L. 403. “While this analysis was only conducted on single-copy core genes, it suggests that some of the unclassified accessory genes (Fig 2 H) may be undergoing similar evolutionary trends.” What exactly is suggesting such a thing? See main comment regarding this. – This sentence has been removed as we did not conduct the analyses to support it’s message.

L. 428. “due to our Illumina based we did not process“ Missing word? Assemblies? – Yes, assemblies was missing.

L. 435-444. This paragraph is about TEs, genome size and gene duplication. The previous one is not, but the one even before is. This makes the reading confusing. Perhaps merge the two related paragraphs together. – We have altered the paragraph orientation to in agreement with the reviewers’ suggestions.

L.442. Typo: “Clavicpes “ - Fixed
