# Supplementary material for: A large accessory genome and high recombination rates may influence global distribution and broad host range of the fungal plant pathogen Claviceps purpurea
Source: PLoS One. 2022 Feb 10;17(2):e0263496. doi: 10.1371/journal.pone.0263496 (PMC8830672; doi:10.1371/journal.pone.0263496)
Supplement: Attachment — Submitted filename: Response to reviewers.docx [file pone.0263496.s016.docx]

We thank the reviewers for their time and input. We have edited the manuscript one more time for grammar and incorporated the recommendations of the reviewers. We reviewed the data again and determined there are 37 missing scaffolds. One of the scaffolds doesn't contain any genes, so we likely did not pick it in our analyses before. We added contig 181 to S6_Table and fixed the 36's back to 37's in the text. The Dryad data has been submitted and is in progress and will be available before publication.
