# Supplementary material for: A large accessory genome and high recombination rates may influence global distribution and broad host range of the fungal plant pathogen Claviceps purpurea
Source: PLoS One. 2022 Feb 10;17(2):e0263496. doi: 10.1371/journal.pone.0263496 (PMC8830672; doi:10.1371/journal.pone.0263496)
Supplement: Attachment — Submitted filename: Response to reviewers.docx [file pone.0263496.s017.docx]

Thank you for catching the last remaining error. I believe I was working of a previous version I had on my computer rather than downloading the file from the editorial center. I believe this was the reason the 3 sentences at L485 were omitted. They have been added and I have reviewed the entire document to ensure their were no other omissions. My apologies.
